# Early Epigenetic Responses in the Genomic DNA Methylation Fingerprints in Cells in Response to Sublethal Exposure of Silver Nanoparticles

**DOI:** 10.3389/fbioe.2022.927036

**Published:** 2022-06-16

**Authors:** Yue Chen, Fei Sheng, Xingyu Wang, Zhihong Zhang, Shiyong Qi, Liqun Chen

**Affiliations:** ^1^ Department of Urology, Tianjin Institute of Urology, The Second Hospital of Tianjin Medical University, Tianjin, China; ^2^ Department of Urology, Nanjing First Hospital, Nanjing Medical University, Nanjing, China; ^3^ Academy of Medical Engineering and Translational Medicine, Tianjin University, Tianjin, China

**Keywords:** silver nanoparticles, epigenetic, DNA methylation, sublethal exposure, cell metabolism

## Abstract

With the rapid development of nanotechnology and nanoscience, nanosafety assessment has raised public concern. Although many studies have illustrated that nanomaterials could lead to genotoxicity, the early alterations of DNA methylation with nanomaterials under low-dose exposure have not been completely clear. In this study, we investigated the potential effect and molecular mechanism of AgNPs on the alternation of DNA methylation fingerprints in HEK293T cells under sublethal exposure. Intriguingly, silver nanoparticle treatment increased 5-mC level and changed methylation-related enzyme contents. Mechanistically, we scrutinized the changes in the molecular signaling and biological functions by means of MeDIP-Seq and RNA-seq. Our results revealed that AgNPs might undermine a number of vital regulatory networks including the metabolic processes, biological regulation and other cellular processes. More specifically at the DNA methylation fingerprints, there were 12 up-regulated and simultaneous hypomethylated genes, and 22 down-regulated and concomitant hypermethylated genes in HEK293T cells responding to AgNPs. Notably, these genes were primarily involved in lipid metabolism and ion metabolism. Together, these responsive genes might be used as early sensitive indicators for the variations of early epigenetic integrity through changing the DNA methylation fingerprints, as reflective of biological risk and toxicity of silver nanoparticles under realistic exposure scenarios.

## Introduction

With the rapid development of nanotechnology and nanoscience, nanosafety assessment has raised public concern regarding their toxic effect on human health. Mounting studies have investigated the genotoxicity of various nanomaterials over the past few years *in vitro* and *in vivo* (Turkez et al., 2017; Elespuru et al., 2018), however, their biological effects on the epigenome remains limited and inconclusive.

Epigenetic alterations are associated with the development and progression of numerous pathological states and diseases (Feinberg, 2007; González-Becerra et al., 2019). As one of the epigenetic mechanisms, DNA methylation is responsible for proper gene expression related with cellular homeostasis in a cell-specific manner (Jones, 2012). Silver nanoparticles (AgNPs), as one of the nanomaterials, have been widely used in production and life due to its inherent properties (Ravikumar et al., 2009). Many studies have been reported to discuss the epigenetic toxicity of AgNPs under toxic doses (Smolkova et al., 2015; Babele et al., 2019). In our previous study, it has been shown that AgNPs induce global genome-wide DNA methylation changes under sublethal dose (Chen et al., 2017). But direct nanotoxicity and indirect impact could inevitably produce distinct toxic or biological effects. Thus, there is a great deal of necessity for explore of epigenetic biomarkers to better identify the early response and molecular mechanisms under sublethal exposure to AgNPs.

In this study, we aimed to investigate the potential effect and molecular mechanism of AgNPs on the alternation of DNA methylation in HEK293T cells under sublethal exposure. Our work illustrated that AgNPs could induce DNA methylation changes and gene expression differences after non-toxic treatment. In addition, we also found AgNPs could alter various cellular processes through influencing the level of DNA methylation in HEK293T cells, such as metabolic process, biological regulation and so on. These findings provided epigenetic reference in evaluating potential toxicity and health risk of AgNPs.

## Materials and Methods

### Characterization of AgNPs

25 nm spherical AgNPs modified by polyvinylpyrrolidone were purchased from Huzheng Nanotechnology Co., Ltd., (Shanghai, China). The AgNPs used in our experiment were kept away from light at 4°C. Transmission electron microscopy (TEM; Hitachi H7500, Japan) was applied to observe the size and shape of AgNPs. The zeta potential and hydrodynamic diameter in water, medium, and complete medium were examined using a Zetasizer (Malvern Nano Series, United Kingdom).

### Cell Culture

Human kidney-derived HEK293T cells were purchased from the Shanghai Cell Bank of Type Culture Collection (Shanghai, China). Cells were cultured in Roswell Park Memorial Institute 1640 medium (C11875500BT; Gibco) contained with 10% fetal bovine serum (10099-141C; Gibco) and 100 U/mL penicillin-streptomycin (SV30010; Hyclone). Cells were cultured in a cell incubator (Thermo Fisher Scientific) at 37°C under humidified air with 5% CO_2_.

### Cell Viability Assessment

Cells were treated with AgNPs at different concentrations (1, 2, 4, 8, 10, 15, 20 μg/ml) for 24 h. Cell viability was assayed by 3-(4,5-dimethylthiazol-2-yl) -2,5-diphenyltetrazolium bromide (MTT) assay using 96-well plates. In brief, HEK293T cells were cultured in 96-well plates and then exposed to AgNPs at different doses for 24 h. After cells were incubated with 10 μL MTT solution (5 mg/ml) for 4 h, 100 μL dimethyl sulfoxide was added into every well. The intensity was detected with a 570 nm absorption wavelength with a microplate reader (Thermo Fisher Scientific).

### Dot-Blotting Assay

Genomic DNA concentrations were quantified by Nanodrop 2000 (Thermo Fisher Scientific, United States). The equal amount of DNA were denatured at 95°C for 10 min, and twofold serial dilutions of DNA were spotted on the nitrocellulose membranes soaked with 2x saline sodium citrate (2x SSC) buffer. Thereafter, the nitrocellulose membranes were baked at 80°C for 2 h, followed by blocking with 5% skimmed milk at room temperature for 1 h. The membranes were incubated with anti-5-mC (1:1000, from the Active Motif, United States) at 4°C overnight. After incubation with secondary Ab, autoradiogram images were measured using a ChemiDoc X-ray Raman scattering (XRS) chemiluminescence system (Bio-RAD, United States).

### UPLC-MS/MS Analysis of 5-mC and 5-hmC Levels in Genomic DNA

Genomic DNA was extracted from cells posttreated with AgNPs using a DNA isolation kit (Qiagen, Germany), followed by UPLC-MS/MS analysis as described previously (Chen et al., 2017).

### Genome-Wide Methylated DNA Immunoprecipitation Sequencing

Genome-wide methylated DNA immunoprecipitation sequencing (MeDIP-seq) was performed by the BGI Tech, Inc. (http://www.bgi.com/us/). Genomic DNA for each sample was isolated and purified, and the sample was sonicated into 100–500 bp fragments. DNA-end repair was implemented through 3′-A overhang and ligation of sequencing adaptors. DNA samples were denatured at 95°C for 10 min, and an aliquot (5 µg) of DNA was immunoprecipitated with anti-5-mC Ab at 4°C overnight. The eluted products were separated and the extracted DNA was amplified with PCR. The following process and bioinformatics analysis of the sequencing libraries were performed at the BGI Tech.

### RNA Sequencing Analysis

RNA sequencing (RNA-seq) was carried out by the BGI Tech, Inc. (http://www.bgi.com/us/). Total RNA was extracted in cells treated to AgNPs for RNA-seq analysis. Differentially expressed genes were defined by changes of gene expression of 2-fold greater or lower in AgNPs-exposed cells compared to the control.

### Gene Ontology and Pathway Analysis

Gene ontology was analyzed using quick GO database within the Interpro bioinformatics database (http://www.geneontology.org). The Pathway analysis was performed using the KEGG database (http://www.genome.jp/kegg).

### Cellular Localization of AgNPs by TEM

After treatment with AgNPs for 24 h, cells were collected and washed three times with phosphate buffer saline (PBS), and fixed in 2.5% glutaraldehyde. And the samples were further treated with pure resin and embedded into beem capsules with pure resin. Ultrathin sections (70 nm) were cut and stained with 1% lead citrate and 0.5% uranyl acetate and then observed with a high-resolution transmission electron microscope (JEOL JEM 2010F, Hitachi, Japan).

### Western Blot Analysis

After cells posttreatment were collected, these cells were washed with cold PBS and were then lysed with RIPA lysis buffer containing the protease inhibitor cocktail (Roche, Switzerland). Same amounts of total proteins were subjected to 8%–12% sodium dodecyl sulfate-polyacrylamide gel electrophoresis (SDS-PAGE), followed by Western blotting, as previously described (Chen et al., 2020). Primary Abs were against GAPDH (1:1000, Santa Cruz Biotechnology, United States), DNMT1 (1:1000, Cellular Signaling Technology, United States), DNMT3A (1:1000, Santa Cruz Biotechnology, United States), TET1 (1:1000, GeneTex, United States), TET2 (1:200, Santa Cruz Biotechnology, United States). Autoradiogram signal was examined on a ChemiDoc XRS chemiluminescence system.

### Detection of 8-Oxo-G for DNA Damage

The levels of 8-oxo-7,8-dihydro- 2′-deoxyguanosine (8-oxo-G) were used to evaluate DNA damage. Cell culture medium was seperated after AgNPs treatment for 24 h, and the supernatants after centrifugation were used to the evaluation of 8-oxo-G with a commercial kit (Dongge Biotechnology, China).

### Evaluation of ROS Level

HEK293T cells exposed to AgNPs or Ag ions was incubated with 10 μM dichlorofluorescein diacetate (DCF-DA) in the dark for 30 min. Then cells was washed with PBS three times and DCF fluorescence was detected using a microplate reader. The excitation and emission wavelength were 488 and 525 nm H_2_O_2_ was used as a positive control.

### Statistical Analysis

Data were shown in mean ± standard error. Independent t-test was used to determine the difference between two samples, while one-way ANOVA was for comparing the difference among multiple (>2) samples. *p*-values were used to calculate the statistical significance.

## Results

### Physicochemical Characteristics of AgNPs and Cytotoxicity Evaluation in HEK293T Cells

**FIGURE 1:**
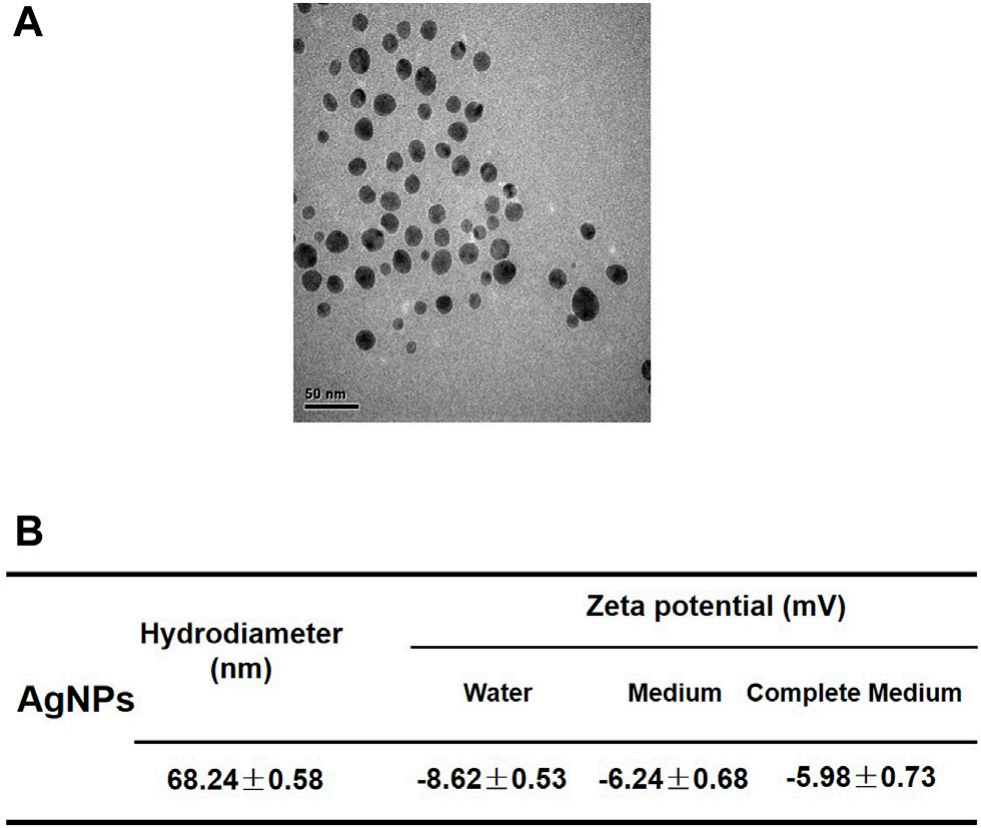
Characterization of silver nanoparticles. **(A)** Representative TEM image of AgNPs applied in this study. The scale bar is 50 nm. **(B)** Hydrodynamic diameter and zeta potentials of 4 μg/ml AgNPs in water, serum-free medium and complete culture medium.

The AgNPs used in our study revealed a spherical structure and well dispersed observed by TEM imaging (Figure 1A). The hydrodynamic diameter of AgNPs was about 68 nm in the medium and the zeta potential of AgNPs are negatively charged in different mediums (Figure 1B). These results indicated that the AgNPs had relatively stable physicochemical property for the following researches.

**FIGURE 2:**
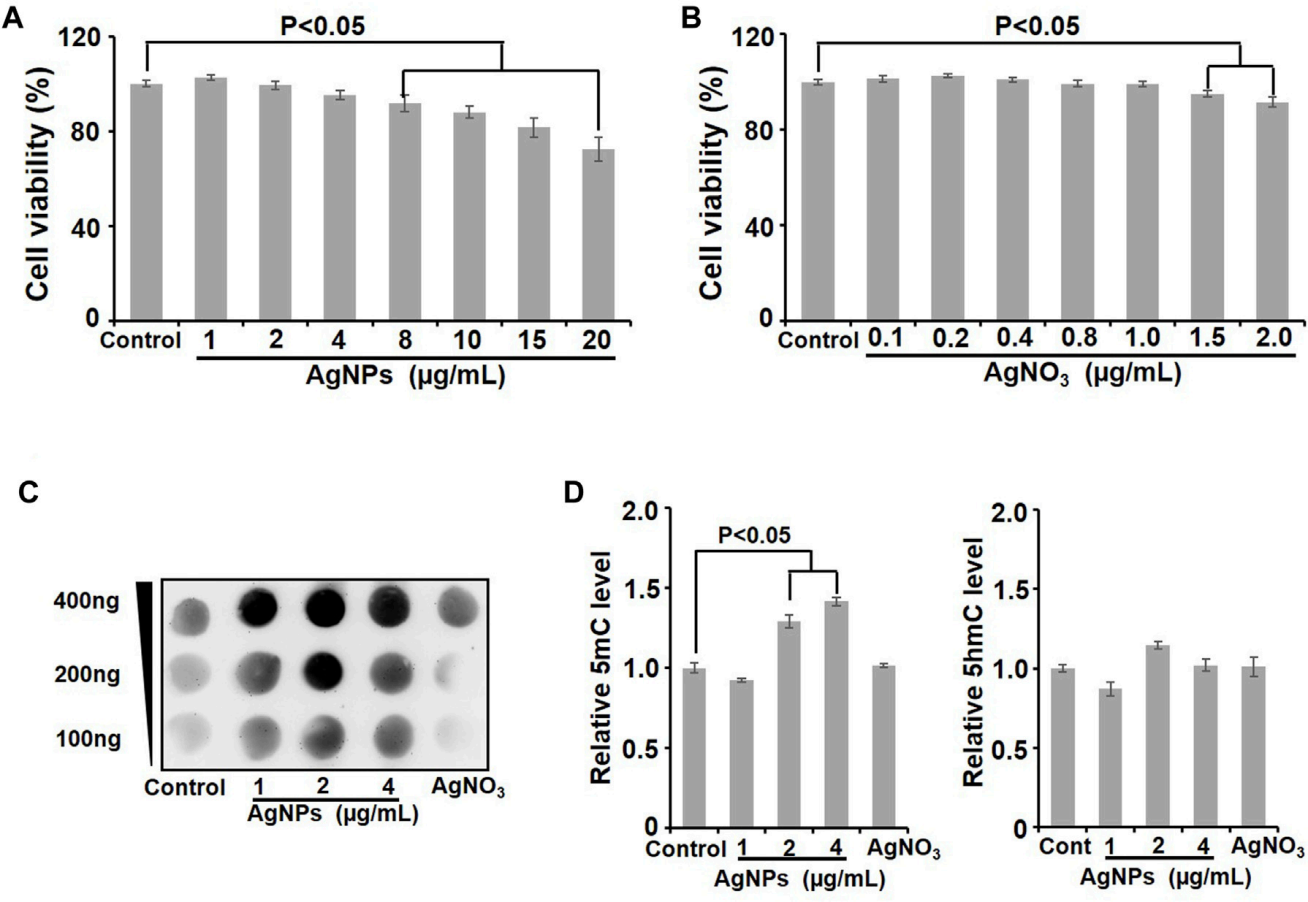
Cell viability evaluation and DNA methylation detection in HEK293T cells after AgNPs exposure. **(A)** Cell viability was detected with the Alamar Blue assay in HEK293T cells exposed to AgNPs at different concentrations for 24 h (*n* = 6). **(B)** Cytotoxicity was examined in HEK293T cells dealt with Ag ions at various concentrations for 24 h through the Alamar Blue assay. **(C)** Dot-blotting analysis of 5-mC values in HEK293T cells upon exposure to AgNPs at different concentrations for 24 h. **(D)** Relative quantitative analysis of 5-mC and 5-hmC levels in genomic DNA detected by UPLC-MS/MS in HEK293T cells upon AgNPs exposure at various concentrations for 24 h (n = 3–4).

Considering the essential excretion way of AgNPs through urine, kidney-derived HEK293T cells were used in this study. To select the sublethal dosage of AgNPs that will not cause overt cytotoxicity to HEK293T cells, we first carried out cytotoxicity assessment in cells treated with AgNPs for 24 h through MTT assay. The results showed that there were no significant cytotoxicity (cell viability decreased slightly by less than 10%) in AgNPs-exposed HEK293T cells at 1, 2, and 4 μg/ml, compared with the untreated control cells (Figure 2A). In view of Ag ions released from silver nanoparticles and its possible toxicity in cells (Pratsinis et al., 2013), we examined the cytotoxicity of Ag ion to HEK293T cells. As shown in Figure 2B, Ag ion did not affect cell viability at no more than 1.0 μg/ml according to about 10% dissolution of AgNPs. To sum up, we applied 1, 2, and 4 μg/ml AgNPs to perform the following research.

### Effect and Distribution of DNA Methylation Changes Upon AgNPs in HEK293T Cells

DNA methylation is known as an early sensitive indicator in response to various endogenous and exogenous stimuli including nanomaterials (Borchiellini et al., 2019). The amounts of 5-methylcytosine (5-mC) and 5-hydroxymethylcytosine (5-hmC) reflect the status of DNA methylation (Meng et al., 2015). In order to study the effect of AgNPs on genomic DNA methylation, Dot blotting and ultraperformance liquid chromatography–tandem mass spectrometry (UPLC-MS/MS) were used to measure the levels of 5-mC and 5-hmC. First, the dot-blotting results demonstrated significant increases of the 5-mC level in AgNPs-exposed cells, especially at 2, and 4 μg/ml, while Ag ion treatment did not cause apparent changes (Figure 2C). Analogous to the results from the Dot blotting assay, the quantitative analysis of UPLC-MS/MS also revealed the similar changes of 5-mC and 5-hmC levels upon AgNPs exposure, compare to the control (Figure 2D). As depicted in bar graph, 5-mC significantly increased in HEK293T cells, particularly at 2 μg/ml and 4 μg/ml respectively (*p* < 0.05). Differently, AgNPs resulted in no obvious changes of 5-hmC in HEK293T cells (Figure 2D). These results together validated that sublethal AgNPs exposure promoted the 5-mC levels in genome-wide DNA in HEK293T cells.

**FIGURE 3:**
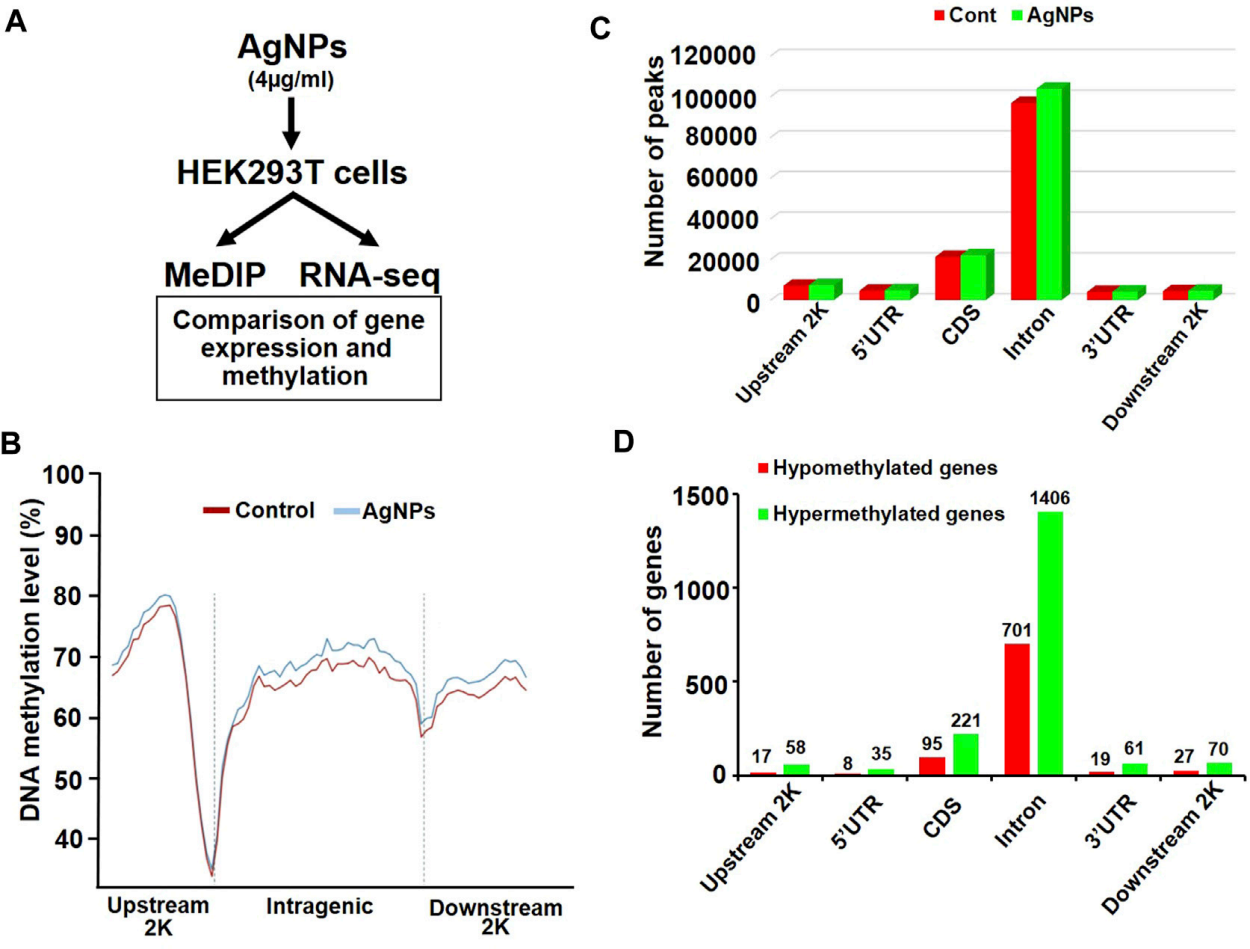
The changes of DNA methylation in different genomic regions in HEK293T cells after AgNPs exposure. **(A)** Simple experimental flow diagram for the sequencing of gene methylation and expression in HEK293T cells exposed to 4 μg/ml AgNPs. **(B)** Relative DNA methylation levels in HEK293T cells treated with AgNPs. **(C)** Distribution of DNA methylation peaks in different genomic regions after AgNPs exposure in HEK293T cells. **(D)** Distribution of hypermethylated and hypomethylated genes in different genomic regions in HEK293T cells treated with AgNPs.

As illustrated in sketch diagram, to investigate the molecular mechanisms of DNA methylation in HEK293T cells exposed to AgNPs, we continued to perform genome-wide methylated DNA immunoprecipitation sequencing (MeDIP-Seq) and RNA sequencing (RNA-seq) to evaluate the property of differentially methylated gene and differentially expressed gene in cells responding to 4 μg/ml AgNPs (Figure 3A). MeDIP-Seq results showed that the genomic DNA methylation level was higher in cells treated with AgNPs than in untreated control, which was consistent with the results from Dot boltting and UPLC-MS/MS assay (Figure 3B). Additionally, levels of DNA methylation were all elevated in upstream, intragenic regions and downstream regions, which suggested the distribution of DNA hypermethylation was a universal increase in the genome (Figure 3B). The methylation distribution analysis manifested that the peaks was mainly increased in the CDS regions and in introns regions (Figure 3C). Subsequently, to define the distribution of abnormal methylated genes in the genome, hypermethylated genes and hypomethylated genes were classified in different regions (Figure 3D). As shown in Figure 3D, the number of hypermethylated genes was two to three times greater than that of hypomethylated genes in AgNPs-exposed cells. In addition, a majority of methylation changes were located in CDS and introns of genes in HEK293T in response to AgNPs (Figure 3D), consistent with the results of 5-mC peak distribution. Together, above results explained the distribution pattern of DNA methylation changes in HEK293T cells upon AgNPs exposure.

### Ontology Analysis of Differentially Expressed Genes in HEK293T Cells Upon AgNPs Treatment

**FIGURE 4:**
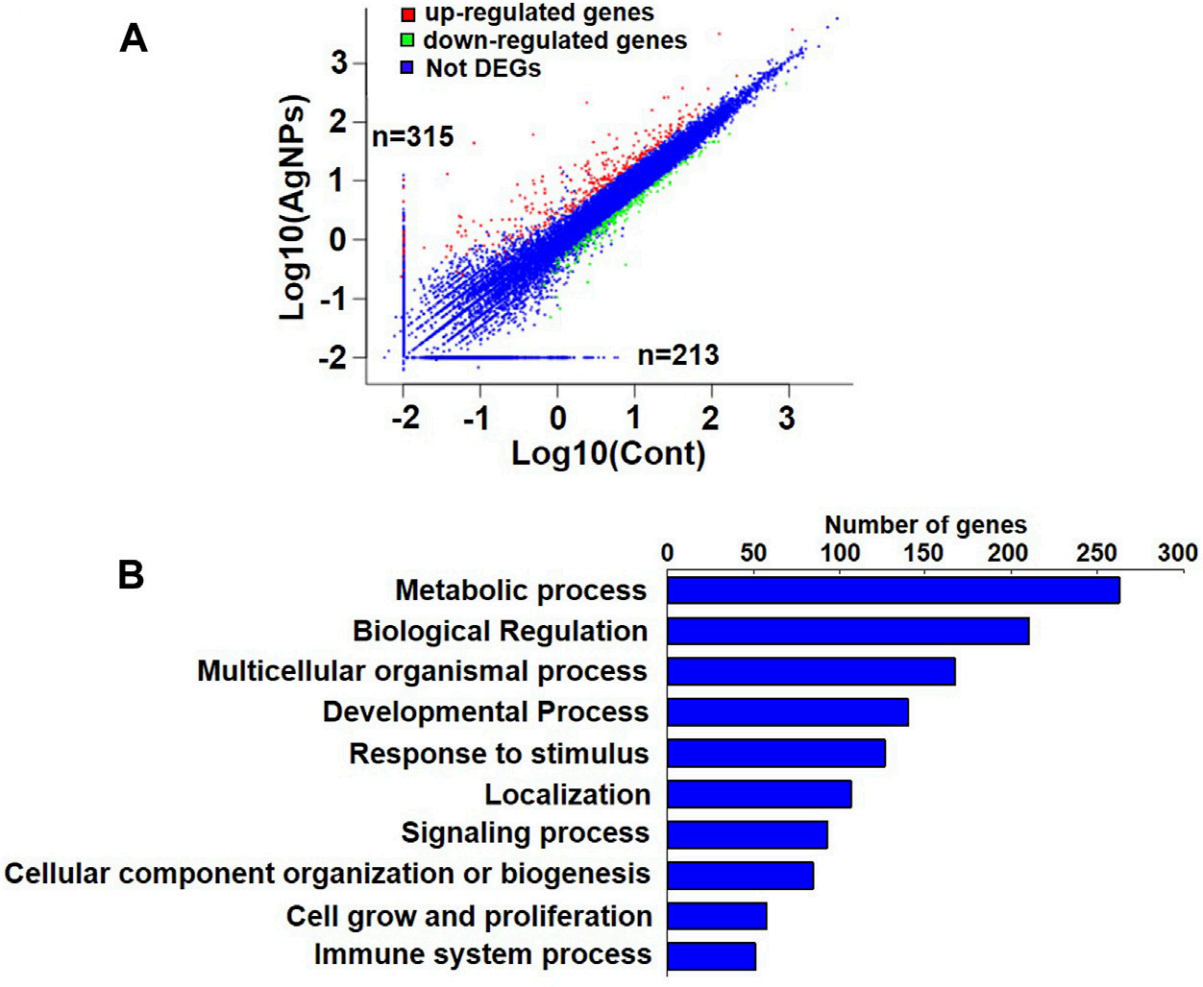
The analysis of gene expression and gene ontology in HEK293T cells exposed to AgNPs. **(A)** Analysis of upregulated and downregulated genes in HEK293T cells upon 4 μg/ml AgNPs exposure. **(B)** Gene ontology and pathway analysis of differential expressed genes in HEK293T cells treated with 4 μg/ml AgNPs.

**FIGURE 5:**
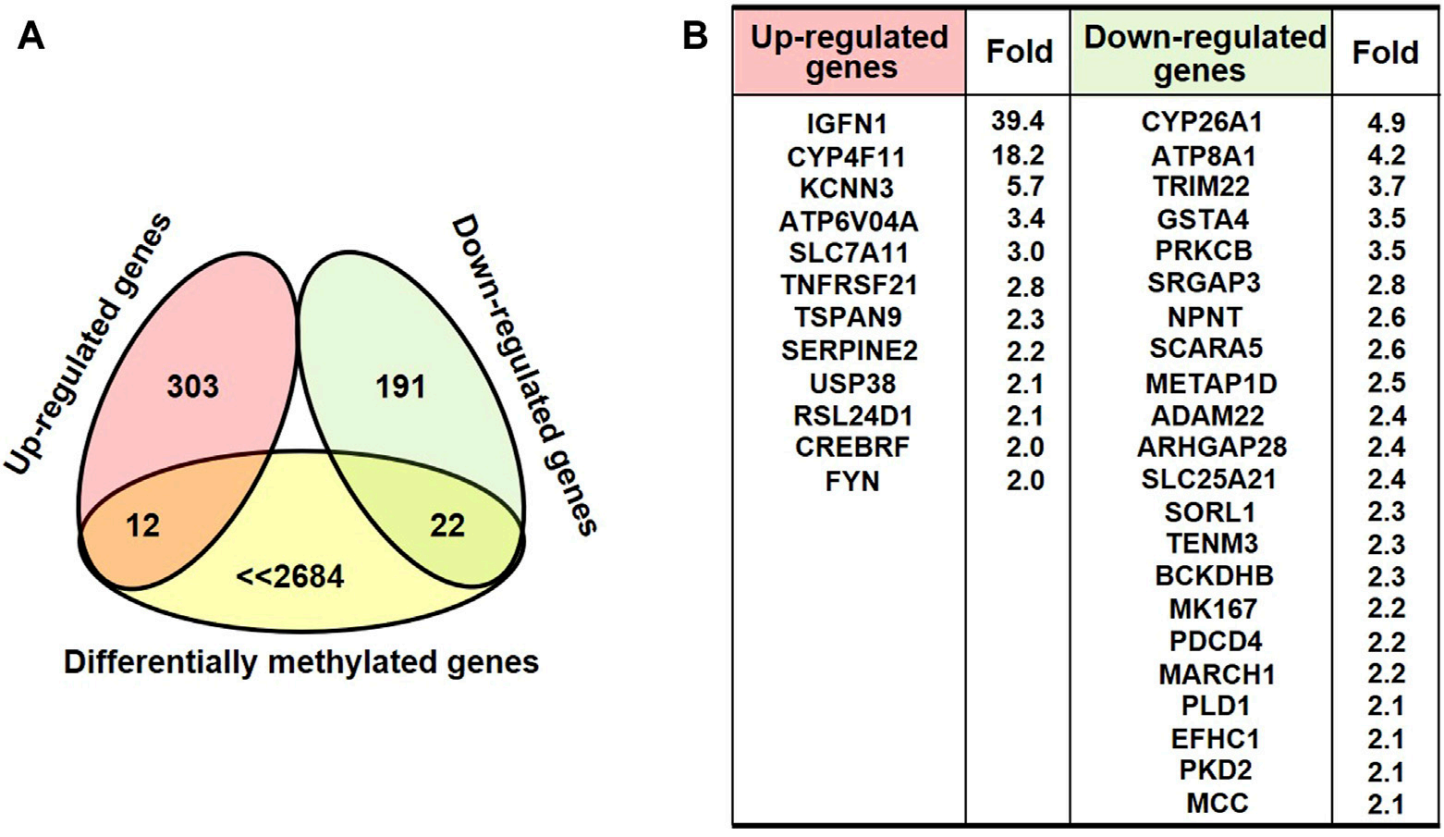
The analysis of differential expressed and methylated genes in HEK293T cells treated with AgNPs. **(A)** The Venn diagram illustrating the differential expressed genes with relevant methylation change in HEK293T cells upon AgNPs. **(B)** The list and the fold for the differential expressed and methylated genes.

DNA methylation changes frequently drove the gene abnormal expression, which was involved in a series of biological process. To identify the possible differential expression in gene transcription due to DNA methylation changes upon AgNPs exposure, we further carried out RNA sequencing to assess the levels of gene expression. As depicted in Figure 4A, there were 315 up-regulated genes and 213 down-regulated genes in HEK293T cells after 4 μg/ml AgNPs exposure. Then, functional classification for differentially expressed genes were carried out through gene ontology analysis. The analytic results revealed that most differentially expressed genes were involved in various aspects of cell dynamic balance, such as metabolic processes, biological regulation, multicellular organismal process and response to stimulus (Figure 4B). With above findings, we hypothesized these differentially expressed genes might originat from DNA methylation changes in AgNPs-treated cells. Therefore, we further compared differentially expressed genes with differentially methylated genes in cells with AgNPs treatment. In general, DNA methylation increase usually results in inhibited mRNA transcription, while DNA demethylation reversely promotes enhanced gene expression (Meng et al., 2015). The Venn diagram demonstrated that there were 12 both upregulated and hypomethylated genes, and 22 both downregulated and hypermethylated genes in response to AgNPs, respectively (Figure 5A). The gene names and folds for these differentially expressed and methylated genes were listed in Figure 5B. Among the all differentially expressed genes, IGFN1, CYP4F11, KCNN3, CYP26A1, and ATP8A1 were much more significant up or downregulated genes, which mainly participated in cell metabolism processes. For instance, CYP4F11, and CYP26A1 primarily catalyze many reactions involved in drug metabolism, synthesis of cholesterol, steroids and other lipids (Wang et al., 2010; Sun et al., 2015; Qiu et al., 2017). KCNN3 and ATP8A1 are mainly responsible for ions metabolism, including the activation and transport of potassium ion, calcium ion and heavy metal ion (Jing et al., 2019; Rahm et al., 2021). These findings suggested that AgNPs sublethal treatment could result in change of cell metabolism drove by DNA methylation change, including lipid metabolism and ion metabolism.

### Possible Mechanisms of DNA Methylation Changes by AgNPs in HEK293T Cells

**FIGURE 6:**
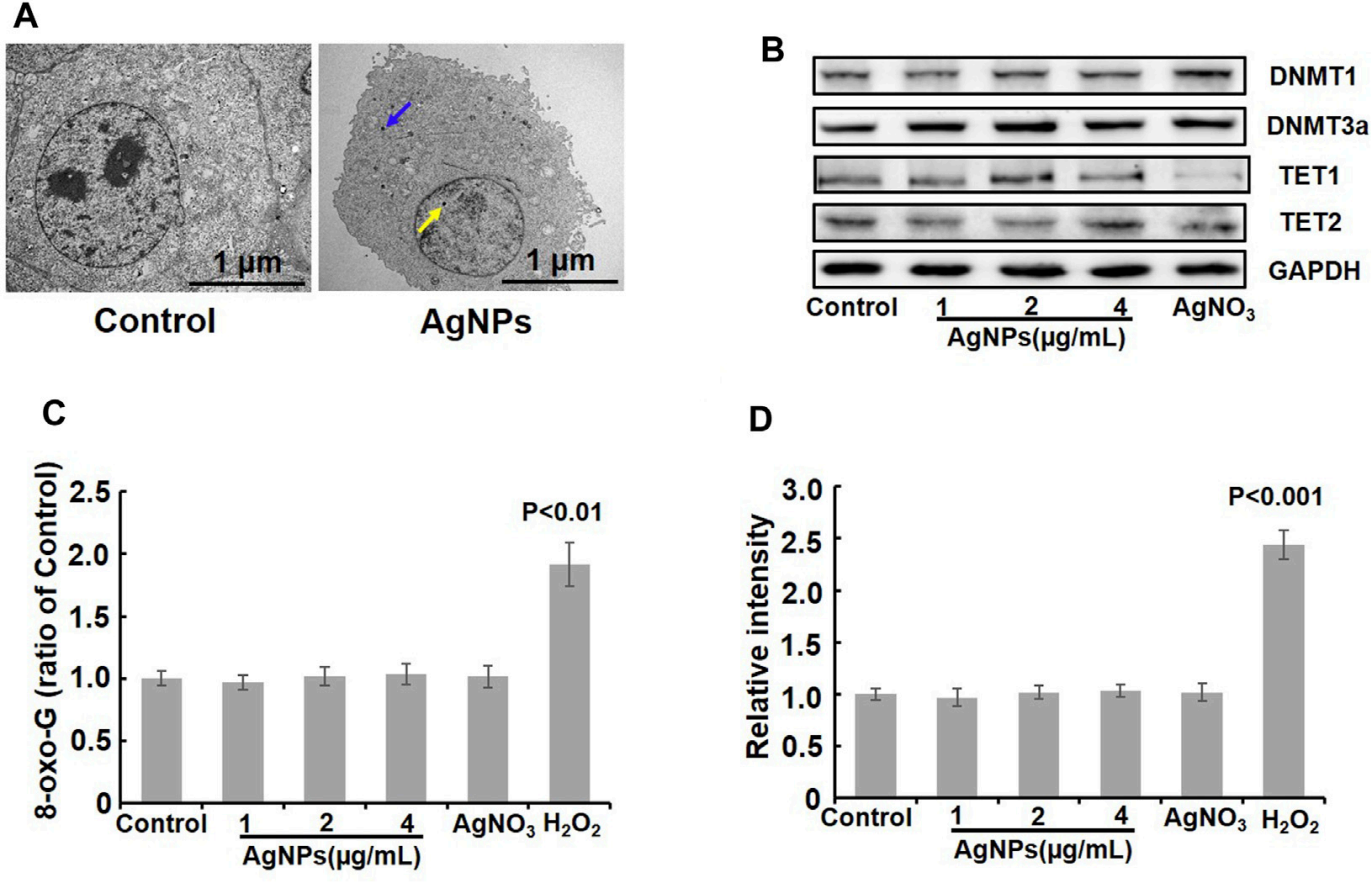
Localization of AgNPs, levels of methylation related enzymes, DNA damage and ROS detection after AgNPs treatment. **(A)** Representative TEM images showing intracellular localization of AgNPs after exposure with 4 μg/ml AgNPs for 24 h. Original magnification ×10,000. **(B)** Western blot analysis of DNMT1, DNMT3a, TET1, and TET2 in HEK293T cells treated with AgNPs and Ag ions for 24 h. **(C)** Relative 8-oxo-G level was detected in HEK293T cells upon AgNPs and Ag ions for 24 h (*n* = 4). **(D)** Relative total ROS level was examined in HEK293T cells exposed to AgNPs and Ag ions for 24 h (*n* = 4).

Previous studies have confirmed that the effects of nanomaterials are closely associated with their physicochemical properties and subcellular localization (Lew et al., 2018; Ovais et al., 2019). To analyze the possible reasons of DNA methylation change induced by AgNPs in HEK293T cells, we first evaluated the cellular localization of AgNPs. TEM images displayed the intracellular and nuclear localization of AgNPs in HEK293T cells (Figure 6A), consistent with previous work (Chen et al., 2020). DNA methylation and demethylation were mainly conducted by methyltransferases (DNMTs) and ten–eleven translocation (TETs) (Meng et al., 2015). Thereafter, we detected the protein contents of DNMTs and TETs to define the role of AgNPs in DNA methylation change. As shown in Figure 6B, AgNPs increased the protein levels of DNMT1 at 2 and 4 μg/ml, and there was a slight drop for DNMT1 in HEK293T cells upon AgNPs at 1 μg/ml. Additionally, there was also a moderate increase for DNMT3a upon AgNPs at all three concentrations. By contrast, the concentration of TET1 and TET2 in HEK293T cells by AgNPs did not manifest a clear decline or increase in a dose-dependent manner. These results suggested that AgNPs-mediated DNA hypermethylation might be partially attributed to the expression levels of DNA methylation related enzymes caused by AgNPs. In view of the impact of DNA damage in epigenetic modification, we examined the production of 8-oxo-G in cells treated with AgNPs. The results indicated that no DNA oxidative damage was observed in cells upon AgNPs exposure at 1, 2,and 4 μg/ml, compared with the control cells (Figure 6C). Oxidative stress is one of the common mechanisms for AgNPs-mediated effects (Hou et al., 2021). Accordingly, we assayed intracellular ROS level in cells exposed to AgNPs. No obvious ROS was induced in cells response to AgNPs compared to the control cells (Figure 6D). These results indicated that sublethal AgNPs-caused DNA methylation change was not on account of DNA oxidative damage and oxidative stress.

## Discussion

In recent years, AgNPs has been gradually applied in the field of biomedical science with the development of nanotechnology and nanomedicine (Chen et al., 2018). In addition to antibacterial effect, the toxic effects of AgNPs have attracted extensive attention, regardless of direct or indirect toxicity. Many studies explored the cellular injury and toxicity of AgNPs under higher exposure dose (Mao et al., 2016; Riaz Ahmed et al., 2017). However, the toxic mechanisms of AgNPs remain incompletely clear, particularly under chronic sublethal dose exposure. Moreover, traditional toxicity evaluation assays can not take effect at sublethal or relatively low dose. Thus, searching for early warning biomarkers are urgently needed to verify the potential toxic effects of low dose AgNPs exposure.

DNA methylation is one of the most intensely studied epigenetic modifications in mammals. In normal cells, it assures the proper regulation of gene expression and stable gene silencing (Kulis and Esteller, 2010). Different from DNA damage, DNA methylation is a more sensitive index in response to external stimuli (Sriraman et al., 2020). Although many reports have illustrated that nanomaterials could lead to genotoxicity, the alterations of DNA methylation with nanomaterials under low dose exposure have not been fully understood (Chen et al., 2017). At present, our previous and some preliminary studies suggested that AgNPs and other nanomaterials might affect DNA methylation (Chen et al., 2017). In the current study, we discussed deeply the potential molecular mechanism of DNA methylation change in HEK293T cells treated with AgNPs under sublethal exposure. Except for the role in the alternation of DNA methylation, we also revealed the changes of molecular signaling and function by means of MeDIP-Seq and RNA-seq under AgNPs sublethal exposure. Importantly, the study found that AgNPs might influence the metabolic process, biological regulation and other cellular processes based on the role of these differentially expressed genes in keeping cell homeostasis.

According to the integrated results from MeDIP-Seq and RNA-seq, there were 12 upregulated and simultaneous hypomethylated genes, and 22 downregulated and concomitant hypermethylated genes in response to AgNPs, which suggested the consistency between gene expression and DNA methylation. Of course, there might be some genes that were not consistently changed at the mRNA level compared with DNA methylation or demethylation. We found that some more obviously upregulated or downregulated genes (including IGFN1, CYP4F11, KCNN3, CYP26A1, and ATP8A1) consistent with DNA methylation change mainly participated in lipid metabolism and ion metabolism. These epigenetic related molecules could be regarded as early warning biomarker to evaluate the potential toxic effects of low dose AgNPs exposure.

Additionally, our study showed intracellular and nuclear localization of AgNPs, elevated protein levels of DNMT1 and DNMT3a and slightly decreased concentration of TET2 in HEK293T cells, conforming to the findings of 5-mC and 5-hmC. We also confirmed that sublethal AgNPs treatment resulted in no significant changes in DNA damage and ROS production, which consistent with previous study at similar exposure concentrations of AgNPs (Chen et al., 2020). Above findings indicated that sublethal AgNPs-mediated DNA methylation change could be partially attributed to DNA methylation related enzymes, independent of DNA damage and oxidative stress.

## Conclusion

In a word, our research clarified the molecular mechanisms responsible for AgNPs-induced DNA methylation alterations. Furthermore, our findings deciphered in-depth the alteration of cell metabolism and related signaling induced by AgNPs due to DNA methylation or demethylation through MeDIP-Seq and RNA-seq, which reflected the potential cytotoxicity and early compensatory responses for AgNPs low dose exposure. The variations of epigenetic and related metabolic biomarkers responding to AgNPs could be used as an early and sensitive indicators to warn possible toxicity or biological effect of nanomaterials. Of course, there may be other biomarker overlooked in this research. In addition, the similarities and differences in distinct nanomaterials or cells still need further investigation.

## Data Availability

The original contributions presented in the study are included in the article/supplementary material, further inquiries can be directed to the corresponding authors.

## References

[B1] Babele P. K. Singh A. K. Srivastava A. (2019). Bio-Inspired Silver Nanoparticles Impose Metabolic and Epigenetic Toxicity to *Saccharomyces cerevisiae* . Front. Pharmacol. 10, 1016. 10.3389/fphar.2019.01016 31572189 PMC6751407

[B2] Borchiellini M. Ummarino S. Di Ruscio A. (2019). The Bright and Dark Side of DNA Methylation: A Matter of Balance. Cells 8 (10), 1243. 10.3390/cells8101243 PMC6830319 31614870

[B3] Chen Y. Wang M. Zhang T. Du E. Liu Y. Qi S. (2018). Autophagic Effects and Mechanisms of Silver Nanoparticles in Renal Cells under Low Dose Exposure. Ecotoxicol. Environ. Saf. 166, 71–77. 10.1016/j.ecoenv.2018.09.070 30248563

[B4] Chen Y. Xu M. Zhang J. Ma J. Gao M. Zhang Z. (2017). Genome-Wide DNA Methylation Variations upon Exposure to Engineered Nanomaterials and Their Implications in Nanosafety Assessment. Adv. Mat. 29 (6), 1604580. 10.1002/adma.201604580 27918113

[B5] Chen Y. Yang T. Chen S. Qi S. Zhang Z. Xu Y. (2020). Silver Nanoparticles Regulate Autophagy through Lysosome Injury and Cell Hypoxia in Prostate Cancer Cells. J. Biochem. Mol. Toxicol. 34 (5), e22474. 10.1002/jbt.22474 32043710

[B6] Elespuru R. Pfuhler S. Aardema M. J. Chen T. Doak S. H. Doherty A. (2018). Genotoxicity Assessment of Nanomaterials: Recommendations on Best Practices, Assays, and Methods. Toxicol. Sci. 164 (2), 391–416. 10.1093/toxsci/kfy100 29701824

[B7] Feinberg A. P. (2007). Phenotypic Plasticity and the Epigenetics of Human Disease. Nature 447 (7143), 433–440. 10.1038/nature05919 17522677

[B8] González-Becerra K. Ramos-Lopez O. Barrón-Cabrera E. Riezu-Boj J. I. Milagro F. I. Martínez-López E. (2019). Fatty Acids, Epigenetic Mechanisms and Chronic Diseases: a Systematic Review. Lipids Health Dis. 18 (1), 178. 10.1186/s12944-019-1120-6 31615571 PMC6792183

[B9] Hou J. Zhao L. Tang H. He X. Ye G. Shi F. (2021). Silver Nanoparticles Induced Oxidative Stress and Mitochondrial Injuries Mediated Autophagy in HC11 Cells through Akt/AMPK/mTOR Pathway. Biol. Trace Elem. Res. 199 (3), 1062–1073. 10.1007/s12011-020-02212-w 32666434

[B10] Jing W. Yabas M. Bröer A. Coupland L. Gardiner E. E. Enders A. (2019). Calpain Cleaves Phospholipid Flippase ATP8A1 during Apoptosis in Platelets. Blood Adv. 3 (3), 219–229. 10.1182/bloodadvances.2018023473 30674456 PMC6373741

[B11] Jones P. A. (2012). Functions of DNA Methylation: Islands, Start Sites, Gene Bodies and beyond. Nat. Rev. Genet. 13 (7), 484–492. 10.1038/nrg3230 22641018

[B12] Kulis M. Esteller M. (2010). DNA Methylation and Cancer. Adv. Genet. 70, 27–56. 10.1016/B978-0-12-380866-0.60002-2 20920744

[B13] Lew T. T. S. Wong M. H. Kwak S.-Y. Sinclair R. Koman V. B. Strano M. S. (2018). Rational Design Principles for the Transport and Subcellular Distribution of Nanomaterials into Plant Protoplasts. Small 14 (44), 1802086. 10.1002/smll.201802086 30191658

[B14] Mao B.-H. Tsai J.-C. Chen C.-W. Yan S.-J. Wang Y.-J. (2016). Mechanisms of Silver Nanoparticle-Induced Toxicity and Important Role of Autophagy. Nanotoxicology 10 (8), 1021–1040. 10.1080/17435390.2016.1189614 27240148

[B15] Meng H. Cao Y. Qin J. Song X. Zhang Q. Shi Y. (2015). DNA Methylation, its Mediators and Genome Integrity. Int. J. Biol. Sci. 11 (5), 604–617. 10.7150/ijbs.11218 25892967 PMC4400391

[B16] Ovais M. Guo M. Chen C. (2019). Tailoring Nanomaterials for Targeting Tumor‐Associated Macrophages. Adv. Mat. 31 (19), 1808303. 10.1002/adma.201808303 30883982

[B17] Pratsinis A. Hervella P. Leroux J.-C. Pratsinis S. E. Sotiriou G. A. (2013). Toxicity of Silver Nanoparticles in Macrophages. Small 9 (15), 2576–2584. 10.1002/smll.201202120 23418027

[B18] Qiu L. Yin R. X. Khounphinith E. Li K. G. Wang D. S. Wu J. Z. (2017). Association of the CYP26A1 Rs2068888 Polymorphism and Serum Lipid Traits in the Chinese Maonan and Han Populations. Int. J. Clin. Exp. Pathol. 10 (12), 11867–11879. 31966551 PMC6966032

[B19] Rahm A.-K. Wieder T. Gramlich D. Müller M. E. Wunsch M. N. El Tahry F. A. (2021). HDAC2-dependent Remodeling of K_Ca_2.2 (*KCNN2*) and K_Ca_2.3 (*KCNN3*) K^+^ Channels in Atrial Fibrillation with Concomitant Heart Failure. Life Sci. 266, 118892. 10.1016/j.lfs.2020.118892 33310041

[B20] Ravikumar B. Futter M. Jahreiss L. Korolchuk V. I. Lichtenberg M. Luo S. (2009). Mammalian Macroautophagy at a Glance. J. Cell Sci. 122 (Pt 11), 1707–1711. 10.1242/jcs.031773 19461070 PMC2684830

[B21] Riaz Ahmed K. B. Nagy A. M. Brown R. P. Zhang Q. Malghan S. G. Goering P. L. (2017). Silver Nanoparticles: Significance of Physicochemical Properties and Assay Interference on the Interpretation of *In Vitro* Cytotoxicity Studies. Toxicol. Vitro 38, 179–192. 10.1016/j.tiv.2016.10.012 27816503

[B22] Smolkova B. El Yamani N. Collins A. R. Gutleb A. C. Dusinska M. (2015). Nanoparticles in Food. Epigenetic Changes Induced by Nanomaterials and Possible Impact on Health. Food Chem. Toxicol. 77, 64–73. 10.1016/j.fct.2014.12.015 25554528

[B23] Sriraman A. Debnath T. K. Xhemalce B. Miller K. M. (2020). Making it or Breaking it: DNA Methylation and Genome Integrity. Essays Biochem. 64 (5), 687–703. 10.1042/EBC20200009 32808652 PMC7606623

[B24] Sun B. Liu K. Han J. Zhao L.-y. Su X. Lin B. (2015). Design, Synthesis, and Biological Evaluation of Amide Imidazole Derivatives as Novel Metabolic Enzyme CYP26A1 Inhibitors. Bioorg. Med. Chem. 23 (20), 6763–6773. 10.1016/j.bmc.2015.08.019 26365710

[B25] Turkez H. Arslan M. E. Ozdemir O. (2017). Genotoxicity Testing: Progress and Prospects for the Next Decade. Expert Opin. Drug Metabolism Toxicol. 13 (10), 1089–1098. 10.1080/17425255.2017.1375097 28889778

[B26] Wang Y. Bell J. C. Keeney D. S. Strobel H. W. (2010). Gene Regulation of CYP4F11 in Human Keratinocyte HaCaT Cells. Drug Metab. Dispos. 38 (1), 100–107. 10.1124/dmd.109.029025 19812349 PMC2802424

